# Exploring the Effects of Omega-3 and Omega-6 Fatty Acids on Allergy Using a HEK-Blue Cell Line

**DOI:** 10.3390/ijms17020220

**Published:** 2016-02-06

**Authors:** Nayyar Ahmed, Colin J. Barrow, Cenk Suphioglu

**Affiliations:** 1 NeuroAllergy Research Laboratory (NARL), School of Life and Environmental Sciences, Faculty of Science, Engineering and Built Environment, Deakin University, 75 Pigdons Road, Waurn Ponds, VIC 3216, Australia; nayyar.khokhar@gmail.com; 2 Centre for Chemistry and Biotechnology, School of Life and Environmental Sciences, Faculty of Science, Engineering and Built Environment, Deakin University, 75 Pigdons Road, Geelong, VIC 3216, Australia; colin.barrow@deakin.edu.au

**Keywords:** fatty acids, atopy, cytokines, IL-4 receptor, IL-13 receptor, STAT6 pathway, IgE antibody, SEAP, HEK-Blue

## Abstract

Background: Allergic reactions can result in life-threatening situations resulting in high economic costs and morbidity. Therefore, more effective reagents are needed for allergy treatment. A causal relationship has been suggested to exist between the intake of omega-3/6 fatty acids, such as docosahexanoic acid (DHA), eicosapentanoic acid (EPA), docosapentanoic acid (DPA) and arachidonic acid (AA), and atopic individuals suffering from allergies. In allergic cascades, the hallmark cytokine IL-4 bind to IL-4 receptor (IL-4R) and IL-13 binds to IL-13 receptor (IL-13R), this activates the STAT6 phosphorylation pathway leading to gene activation of allergen-specific IgE antibody production by B cells. The overall aim of this study was to characterize omega-3/6 fatty acids and their effects on STAT6 signaling pathway that results in IgE production in allergic individuals. Methods: The fatty acids were tested *in vitro* with a HEK-Blue IL-4/IL-13 reporter cell line model, transfected with a reporter gene that produces an enzyme, secreted embryonic alkaline phosphatase (SEAP). SEAP acts as a substitute to IgE when cells are stimulated with bioactive cytokines IL-4 and/or IL-13. Results: We have successfully used DHA, EPA and DPA in our studies that demonstrated a decrease in SEAP secretion, as opposed to an increase in SEAP secretion with AA treatment. A statistical Student’s *t*-test revealed the significance of the results, confirming our initial hypothesis. Conclusion: We have successfully identified and characterised DHA, EPA, DPA and AA in our allergy model. While AA was a potent stimulator, DHA, EPA and DPA were potential inhibitors of IL-4R/IL-13R signalling, which regulates the STAT6 induced pathway in allergic cascades. Such findings are significant in the future design of dietary therapeutics for the treatment of allergies.

## 1. Introduction

Over the past few decades, there has been a dramatic rise in allergic individuals worldwide. An increased level of allergy is leading to increased financial burdens for governments and healthcare organizations, in addition to causing medical concern [1,2]. Allergic reactions can be as mild as a sneeze or in some cases life-threatening, such as an anaphylactic shock [3]. Many studies have been carried out to investigate the causal relationship between allergy and various factors that result in atopic disease, such as genetic predisposition, lifestyle and/or environment [4,5]. The occurrence of allergic diseases has become more prevalent in the industrialized world. There has been a marked increase in prevalence of atopic disease in regions such as Western Europe, the US and Australasia during recent years. A 20-fold increase has been experienced by western countries and as much as 40% of the population suffers from this disease [6]. However, the results are skewed and more research is needed to enhance understanding of exactly how fatty acids interact with different cells to stimulate or inhibit the effect of allergy. Although it is known that allergies run in families, and that genetic predisposition is the largest factor, dietary factors have also become very important in recent times [3]. The intake of certain fatty acids at a young age and their role in determining the outcome of atopic disease in later life has been thoroughly investigated by many scientists throughout the world, via clinical and non-clinical studies [7,8,9,10,11,12].

It has been suggested that an imbalance in dietary intake of essential fatty acids (EFA) such as omega-3 poly-unsaturated fatty acids (n-3 PUFA) and omega-6 polyunsaturated fatty acids (n-6 PUFA) may lead to a predisposition to allergic disease. This is usually caused by an increased intake of n-6 fatty acids, such as linoleic acid (LA, C18:2), with a simultaneously decreased intake of omega-3 fatty acids, such as docosahexanoic acid (DHA C22:6), eicosapentanoic acid (EPA C20:5) and docosapentanoic acid (DPA C22:5) [13]. Such an imbalanced diet leads to an insufficiently balanced T helper cell Types 1 and 2 (Th1 and Th2) pathways. Evidently, high cell membrane levels of arachidonic acid (AA, C20:4n-6) stimulate a Th2 differentiation of naïve T cells, whereas membrane bound n-3 PUFA can stimulate the Th1 variant [13]. In atopic individuals, an immune response is mounted by T cells that are activated by allergens, promoting T helper Type 2 (Th2) variant of cells. Once stimulated, these cells subsequently produce cytokines such as interleukin-4 (IL-4), interleukin-5 (IL-5) and interleukin-13 (IL-13), which stimulate B lymphocytes to produce immunoglobulin E (IgE) antibodies. Subsequently, this reaction triggers the release of histamines and leukotrienes that result in allergic symptoms [3,14]. Unlike the cytokines produced by T helper Type 1 (Th1) cells, such as interferon-gamma (IFN-γ) and interleukin-2 (IL-2) in non-atopic individuals, an excessive production of cytokines takes place from Th2 cells [15].The shift in cytokine production from Th1 cells (IFN-γ and IL-2) to Th2 (IL-4, IL-5 and IL-13) variant forms a hallmark of allergic response in atopic individuals [15].

It was recently reported in dietary intervention studies that during pregnancy n-3 PUFA show a strong tendency to reduce serum IgE antibody levels. In a different study carried out by Weise *et al.* [16]. B cells treated with docosahexanoic acid (DHA) inhibited IgE production by inhibiting the STAT6 phosphorylation pathway [16]. It has also been suggested that DHA can reduce IgE production by human B cells, by affecting the IL-4 pathway via an effect on CD40 ligand [16].

This study along with others has prompted researchers across the world to revisit the effects of fatty acid supplements on allergic individuals. The objective of the current study was to investigate the effect of n-3 and n-6 fatty acids on the STAT6 phosphorylation pathway that leads to the production of IgE antibodies when stimulated by bioactive cytokines such as IL-4 and IL-13. Such findings are significant in the future design of dietary therapeutics for the treatment of allergies.

## 2. Results

### 2.1. HEK-Blue Cell Viability Test Using Cytokines IL-4 and IL-13

**Figure 1:**
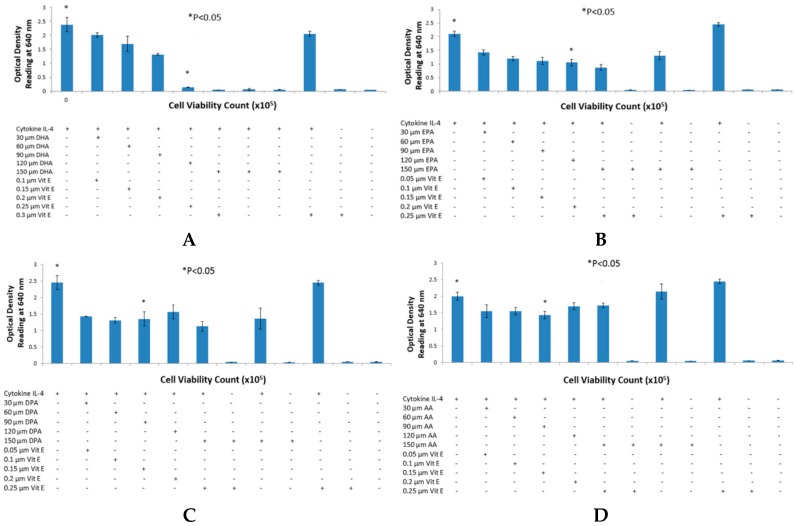
IL-4 detection assay with n-3 and n-6 fatty acids (μM) with HEK-Blue Cells using Quanti-Blue. (**A**,**B**) shows the detection assay performed with docosahexanoic acid (DHA) and eicosapentanoic acid (EPA), respectively, with a dose-dependent inhibition; However in (**C**,**D**), a dose-dependent inhibition does not occur with docosapentanoic acid (DPA) and arachidonic acid (AA) but rather a dose-response inhibition occurs. n-3 fatty acids showed no abnormal effects on secreted embryonic alkaline phosphatase (SEAP) secretion without vitamin E. However, AA without vitamin E caused higher than normal SEAP secretion. * *p* < 0.05 denotes significance of optical density (OD) readings compared to positivecontrol.

**Figure 2:**
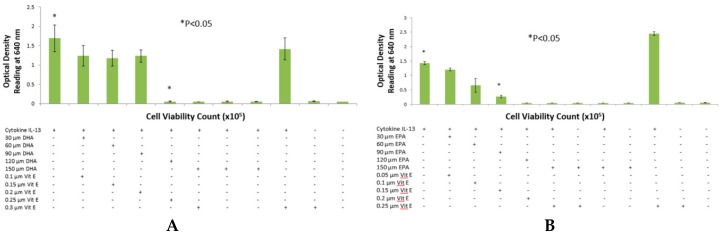

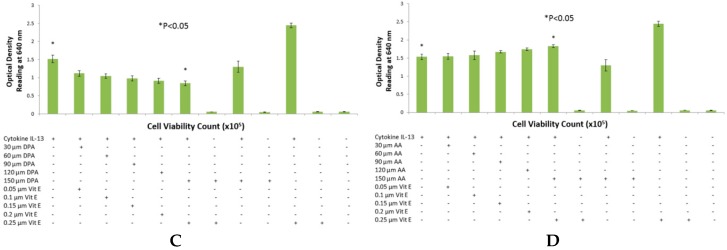
IL-13 detection assay with n-3 and n-6 fatty acids (μM) with HEK-Blue Cells using Quanti-Blue. (**A**) shows a dose-response inhibition of SEAP by DHA; (**B**,**C**)depict the detection assay performed with EPA and DPA, respectively, with a dose-dependent inhibition of SEAP secretion from HEK-Blue cells; However, (**D**) with higher concentrations of AA, there is a rise in SEAP levels hence, and thus a higher optical density reading. n-3 fatty acids showed no abnormal effects on SEAP secretion without vitamin E. However, AA without vitamin E caused higher than SEAP secretion than the positive control. * *p* < 0.05 denotes significance of optical density readings compared to positive control (PC).

A total of two sets with four assays in each set were performed using cytokines IL-4 (Figure 1) and IL-13 (Figure 2). A dose-dependent relationship was observed between fatty acid concentration and secreted embryonic alkaline phosphatase (SEAP) secretion from HEK-Blue cells. Blue coloration was reduced with a gradual increase in fatty acid concentration when Quanti-blue was mixed with cell supernatant. All positive and negative controls behaved as expected with high and low peaks as suggested by the HEK-Blue IL-4/IL-13 kit. As hypothesized, statistically significant results were achieved with an optimum concentration of fatty acids at 90 μM for n-3 PUFA. All inhibitions achieved with n-3 fatty acid treatments were statistically significant with *p* values being less than 0.05 with a two-tailed Student’s *t*-test. While DHA (Figure 1A and Figure 2A) and EPA (Figure 1B and Figure 2B) showed gradual decrease in SEAP secretion with both IL-4 and IL-13, DPA (Figure 1C and Figure 2C) showed a sudden drop in SEAP levels suggesting a more potent inhibition. However, the treatment of HEK-Blue cells with n-6 PUFA AA showed no significant change in the IL-4 cytokine assay (Figure 1D). However, an increase in SEAP secretion was observed with AA treatment when testing cells with IL-13 cytokine (Figure 2D). Optical density readings showed no inconsistencies when n-3 fatty acids were incubated with HEK-Blue cells without vitamin E. However, in the case of AA, it was noted that SEAP secretions were higher than the positive control. Consistency was also observed with cell viability (results not shown) when tested for all the treatments and compared to the positive control.

### 2.2. HEK-Blue Time Course with Fatty Acid and Vitamin E Treatment

**Figure 3:**
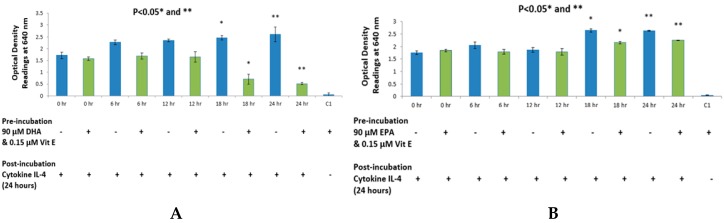

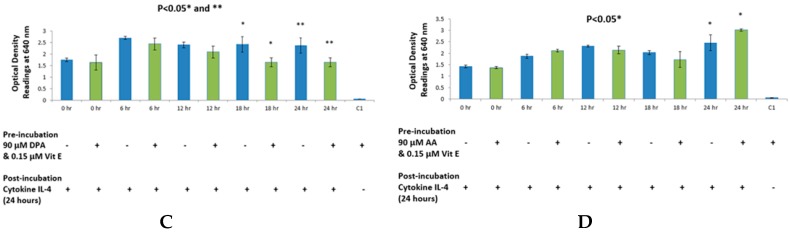
HEK-Blue detection assay with 90 μM of omega-3 and omega-6 fatty acids with 0.15 μM vitamin E using cytokine IL-4 as the bioactive agent. (**A**–**C**) depict a time-dependent inhibition of SEAP secretion from HEK-Blue cells when treated with DHA, EPA and DPA, respectively; (**D**) shows an increase in SEAP secretion from HEK-Blue cells when treated with AA at the 24-h time point. * *p* < 0.05, ** *p* < 0.05 denotes significance of optical density readings compared to positive control depicted in blue colour. Control 1 (C1) depicts the optical density reading obtained when HEK-Blue cells are treated with fatty acid only, without the cytokine stimulation.

**Figure 4:**
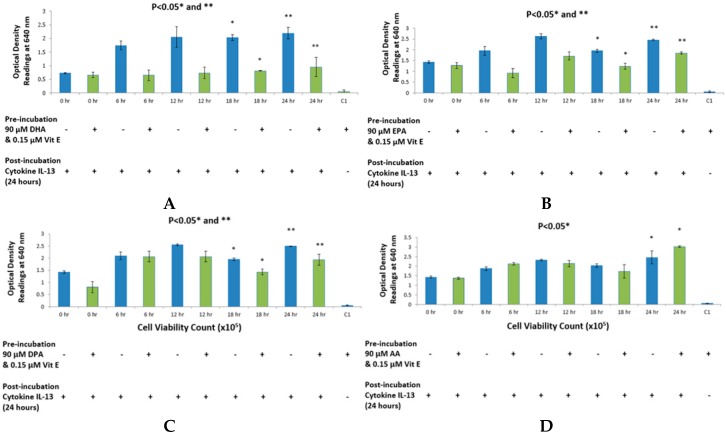
HEK-Blue detection assay with 90 μM of omega-3 and omega-6 fatty acids with 0.15 μM vitamin E using cytokine IL-13 as the bioactive agent. (**A**–**C**) depict a time-dependent inhibition of SEAP secretion from HEK-Blue cells when treated with DHA, EPA and DPA, respectively; Once again, (**D**) shows an increase in SEAP secretion from HEK-Blue cells when treated with AA at the 24-hour time point. * *p* < 0.05, ** *p* < 0.05 denotes significance of optical density readings compared to positive control depicted in blue colour. Control 1 (C1) depicts the optical density reading obtained when HEK-Blue cells are treated with fatty acid only, without cytokine stimulation.

As shown in Figure 3 and Figure 4, a total of eight assays were performed with four assays in each set. Figure 3 shows results with cytokine IL-4 whereas Figure 4 shows results with cytokine IL-13. DHA (Figure 3A and Figure 4A), EPA (Figure 3B and Figure 4B) and DPA (Figure 3C and Figure 4C) all showed a decrease in SEAP secretion and hence produced less blue coloration at 18 and 24 hours’ time-points when compared to the positive control values. All inhibitions achieved with n-3 fatty acid treatments were statistically significant with *p* values being less than 0.05 with a two-tailed Student’s *t*-test. Since the concentration of fatty acid was constant at 90μM, this experiment was a time-dependent inhibition of STAT6 phosphorylation in HEK-Blue cells. However, as seen in Figure 3D and Figure 4D, AA was shown to increase SEAP secretion hence, it shows a higher absorption reading at 18 and 24 hours’ time-points when compared to the positive control values. No significant change was observed at 0, 6 or 12 hours’ time-points. Cell viability was performed at the end of the 24-h incubation to observe anomalies, if any. It was found that AA showed a higher cell count when compared to positive controls in both experiments; with cytokine IL-4 and IL-13. However, the cell viability count remained unaffected with the treatments.

## 3. Discussion

Intake of pro-inflammatory omega-6 fatty acids, such as AA, may have increased in the past few decades with a concurrent decrease in omega-3 fatty acid dietary intake. This has contributed to an increase in allergy prevalence as well as clinical manifestations of allergic disease [17,18,19]. Diets rich in fatty fish will contain relatively high levels of n-3 PUFA, as opposed to n-6 PUFA. N-3 PUFA, particularly DHA, EPA and DPA, exert anti-inflammatory effects, whereas n-6 fatty acids do not [20]. The purpose of this study was to {investigate the effects that n-3 and n-6 fatty acids have on STAT6 phosphorylation pathway, which leads to IgE production in B lymphocytes resulting into allergy. The rationale of the present study was to investigate the authenticity and general observations made in previous studies in the treatment of allergic diseases. We have shown that n-3 fatty acids DHA, EPA and DPA all suppress and down-regulate SEAP secretion in the transfected HEK-Blue cell model hence reducing allergy. The HEK-Blue model uses a very simple colorimetric analysis as an alternative method to measuring IgE antibody production from B cells. Incubation with all three n-3 PUFA resulted in a dose-dependent decrease in SEAP secretion, which in turn gave less blue color when added to the Quant-Blue substrate. This decrease in SEAP secretion was not due to a higher cell proliferation as demonstrated by trypan blue cell viability count. Cell viability count remained unaffected before and after incubation of cells with treatments. The cell count range was noted to hover at a titre of 1 × 10^5^ per treated well. Positive controls were relatively higher in Figure 1 and Figure 2 since fatty acid treatment inhibited STAT6 phosphorylation in HEK-Blue Cells. Nevertheless, the treatment wells were incubated for longer periods when compared to 0 h treatment in which the supernatant from the cells was immediately used for SEAP secretion (pre-incubation period was zero as opposed to 6, 12 h, *etc*.). Hence, the cells were allowed to incubate for longer and had a higher cell count compared to 0 h wells (range remained unchanged at 1 × 10^5^).

The reason for choosing DHA, EPA and DPA for n-3 fatty acid analysis was due to their abundant availability. Breast milk contains all three of these fatty acids and they are also found in a variety of fish and some meat products such as beef and lamb [21]. Secondly, it is well established and documented through clinical studies that DHA, EPA and DPA have a strong link with allergy [22]. Recent publications have reported that lower incidence of eczema was observed in children who consumed fish and whose mothers had higher intake of fish diet during gestation period [23,24]. These studies lead to a possible explanation of reduced SEAP secretion from HEK-Blue cells reducing clinical symptoms in allergic individuals, which may as well be a result of less intake of Omega-3 fatty acids.

In a study carried out by Weise *et al.*, it was found that DHA inhibited IgE production by B lymphocytes, which are derived from human peripheral blood mononuclear cells. So far, the underlying mechanism is unknown. Since this study was already established in the case of DHA, we tested other n-3 PUFA, in addition to DHA, to improve the overall picture of omega-3 and omega-6 fatty acids. It is also known that IL-4R alpha (CD124) is suppressed when B lymphocytes are incubated with n-3 fatty acids, which leads to a suppression of STAT6 phosphorylation pathway [16,20]. As hypothesized, DHA, EPA and DPA all behaved very similarly in the time course experiment in suppressing STAT6 signaling in HEK-Blue cells. The Janus kinase (JAK) signaling molecules activate STAT6 transcription factor that subsequently activates gene expression in the cell nucleus. Expression and activity of protein kinases can be altered by different fatty acids. A study carried out by Van Meter *et al.* (1994) suggests that EPA and DHA can suppress the activity of protein kinase C (PKC), which leads to inhibition of STAT6 dimerization and gene suppression [25,26]. This is a possible explanation for less SEAP production by HEK-Blue cells. Furthermore, DHA did not exhibit a dose-dependent inhibition in the IL-13 detection assay but rather a dose-response inhibition. With the highest concentration of DHA, it was observed that inhibition was the highest. Literature suggests that high dose of DHA supplementation changes the outcome of IgE production from B cells in response to cytokine stimulation, which may explain the sudden drop in SEAP secretion at 150 μM [16,27].

The purpose of choosing AA for these studies was due to the abundance of this fatty acid in body tissues. It is a major component of mammalian cell membranes and accounts up to a quarter of all phospholipid fatty acid [28,29], hence making it a very important target for allergy studies because of its ubiquitous nature. No significant change was observed in the case of AA when using a range of concentrations. However, in the time course experiment, an increase in SEAP production was observed, as well as slightly elevated levels of cell viability counts. This evidence is in agreement with the fact that AA causes cell proliferation in endothelial cells and HEK-Blue cells are derived from endothelial cells found in kidneys [30]. It was also observed that AA incubated with HEK-Blue cells without vitamin E supplementation gave higher SEAP secretion than the positive control. A higher optical density reading was also observed in the case of EPA and DPA when used with IL-4 and DPA when used with IL-13. This is not consistent with literature, which suggests that vitamin E suppresses the production of IgE antibody from B cells and hence, it can suppress SEAP secretion by the same mechanism. In a study carried out by Inagaki *et al.*, vitamin E was supplemented to mice in diets along with oral administration of sesame oil, which resulted in suppression of IgE antibody production [31]. Another clinical trial observed that dietary intake of vitamin E reduced serum IgE concentrations in allergic individuals suffering from atopic dermatitis [32]. However, this was not true in the case of DHA, when used with IL-4 and IL-13, and EPA with IL-13, which suggests that vitamin E may have specific effects on STAT6 phosphorylation in combination with different fatty acids. Furthermore, AA showed inhibition of SEAP secretion with IL-4 detection assay, which is an anomaly we found in the overall effects of AA. This may be explained by the fact that AA incorporated into cell membranes can affect membrane fluidity which may consequently effect cell function [20,33,34].

Another equally important explanation can be the production of inflammatory prostaglandins from HEK-Blue cells. AA is found esterified at the *sn-2* positions of membrane phospholipids. AA can be released by cytoplasmic phospholipase A_2_ and converted by cyclooxygenase (COX) 1 and 2 to prostaglandin intermediates known as PGH_2_. Prostaglandin synthases then convert PGH_2_ to 2-series prostanoids known as prostaglandin E_2_ (PGE_2_), which are inflammatory. AA can also be converted to thromboxanes (TX) of the 2-series for PGD_2_ or by 5-, 12-, and 15-LOX, which yield inflammatory leukotrienes of the 4-series. It was found that AA was suppressing IL-13 stimulation of HEK-Blue cells when tested with higher doses of AA. A study suggests that IL-13 suppresses COX-2 gene expression [35]. By contrast, n-3 PUFA such as DHA and EPA can replace AA in cell membranes and can be converted by the same enzymes to anti-inflammatory intermediates such as the 3-series prostanoids known as prostaglandin E_3_ (PGE_3_) [20,36,37]. In fact, EPA and DHA are poor COX substrates and potent antagonist to COX activity. Also, gene expression of proteins involved in AA metabolism can be suppressed by the action of EPA and DHA [37]. All kidney derived cells are capable of producing the COX series of enzymes hence, incubating cells with fatty acids can alter gene expression through modification of transcription factor activity [38,39]. This may effect SEAP secretion from HEK-Blue cells. Although not part of this study, it is important to mention that PGE_2_ receptors regulate activation and differentiation of mouse B lymphocytes to IgE production [40].

## 4. Materials and Methods

### 4.1. Cell Culture, PUFA and Vitamin E Reagents and Buffers

HEK-Blue™ IL-4/IL-13 (InvivoGen, San Diego, CA, USA) cells allow the detection of bioactive IL-4 and IL-13 signaling by monitoring the activation of the JAK-STAT6 phosphorylation pathway. The cells are a result of a stably transfected human JAK-STAT6 gene. Other pathways linked to the STAT6 pathway are naturally expressed by the cells. The cells are further transfected with a STAT6-inducible SEAP reporter gene, as an alternative to IgE secretion in B lymphocytes, when signaled by cytokines IL-4 or IL-13. Finally, the cells are transfected with a stably expressed recombinant type II IL-4Rα/IL-13αR1 receptor.

Growth medium: HEK-Blue cells were cultured in Dulbecco’s Modified Eagle Medium (DMEM) (*GIBCO*), supplemented with 10% fetal bovine serum (FBS), in a humidified incubator at 37 °C with 5% CO_2_. Antibiotics were added to the medium to avoid contamination (50 U/mL of penicillin, 50 µg/mL of streptomycin, 100 µg/mL of normocin, 10 µg/mL of blasticidin and 100 µg/mL of zeocin-*GIBCO*), Trypsin/EDTA (0.05%; *GIBCO*) was used for trypsinization.

Experimental medium: Growth medium was replaced with 2 mM l-glutamine and heat inactivated 10% FBS along with omega-3 and omega-6 fatty acids; namely, DHA (*M*w 328 Da), EPA (*M*w 302 Da), DPA (*M*w 330.5 Da) and AA (*M*w 304.46 Da), all of which were commercially purchased from Invitrogen (Carlsbad, CA, USA) at a concentration of 10 μg/mL. The final concentrations prepared were 30, 60, 90, 120 and 150 μM using 100% ethanol as the solvent. Vitamin E (Invitrogen, Carlsbad, CA, USA) was prepared as an antioxidant to prevent oxidation of fatty acids using growth medium. The final concentrations prepared were 0.05, 0.1, 0.15, 0.2 and 0.25 μM to all corresponding treatments outlined above.

Initially, cells were tested in the presence of ethanol and vitamin E separately to confirm whether cell viability and/or STAT6 phosphorylation pathway was affected or whether it caused apoptosis in cells. Cells were incubated with experimental medium containing ethanol and vitamin E over a period of 24 h and cell viability was tested at five intervals (0, 6, 12, 18 and 24 h). Our results confirmed that the amounts and concentrations of ethanol and vitamin E used in our study had no effect on cell viability and/or STAT6 phosphorylation pathway with or without the addition of ethanol and vitamin E (results not shown). Cells were then ready to be tested with fatty acids.

Stock solutions of DHA, EPA, DPA and AA in ethanol were stored at −20 °C, and then these PUFA were pre-incubated in complete growth medium at 37 °C overnight to allow protein conjugation. We tested a range of concentrations on HEK-Blue cells to establish that the STAT6 phosphorylation pathway lead to the production of serum embryonic alkaline phosphatase (SEAP). This is an alternative product to IgE antibody that is produced by B lymphocytes when triggered by the bioactive IL-4 or IL-13. SEAP breaks down Quanti-Blue substrate, which gives the blue coloration. The blue color can then be detected using a spectrophotometer. Vitamin E stock was stored at 4 °C and was freshly prepared in ethanol when preparing experimental media with fatty acids.

Two sets of experiments were performed to determine the effects of fatty acids on the STAT6 phosphorylation pathway. In the preliminary experiments, a range of different concentrations of fatty acids and vitamin E were studied to confirm their effect on SEAP production. Once confirmed, the most optimum concentration of fatty acid was used to perform a time course experiment. Cell viability and SEAP production were detected at five different time-points over a period of 24 h. These experiments allowed us to study two different variables; time and concentration of fatty acid needed to suppress SEAP secretion by HEK-Blue cells by using a very simple colorimetric analysis.

### 4.2. HEK-Blue Cell Viability Test Using Cytokines IL-4 and IL-13

The protocol used was taken from the datasheet provided with the HEK-Blue IL-4/IL-13 kit. A few adjustments were made in the protocol to perform the assay without compromising the signalling in cells. Cells were grown in T-75 flasks and used for detection assay. The cells were detached using 3 mL trypsin, which was neutralized with 7 mL of media containing FBS to give a total volume of 10 mL. The average cells were counted using a TC10 automated cell counter (Bio-Rad Laboratories, Inc., Hercules, CA, USA). HEK-Blue cells were pipetted into 15 mL tubes, which were centrifuged at 1200 RPM for 5 min. The supernatant was discarded and experimental media was added to suspend the cell pellet. If required, cell titre was reduced by adding growth medium to achieve optimum cell density. The cells were pipetted into 24-well plates (Corning, Sigma-Aldrich, St. Louis, MO, USA) at a density of ~50,000 cells per well in a total volume of 720 μL. A total of 12 treatment wells were setup in triplicates. Cells were treated with a combination of fatty acids (30, 60, 90, 120 and 150 μM) with and without vitamin E (0.05, 0.1, 0.15, 0.2, 0.25 μM). A total of 80 μL of cytokine IL-4 (Integrated Sciences, Chatswood NSW 2067, Australia) and IL-13 (Integrated Sciences, Chatswood NSW 2067, Australia) were pipetted to each treatment well at a concentration of 100 ng/mL. Positive controls (PC: with cytokine stimulation) and negative controls (NC: without cytokine stimulation) were included to provide a comparative study. Cells were incubated with the treatments for a period of 24 h. Post-incubation, 20 μL of induced HEK-Blue IL-4/IL-13 cells supernatant from each treatment well was added to a corresponding plate with 180 μL of Quanti-Blue substrate in each well. This was incubated for a total of 3 h to detect SEAP levels. The blue color was detected using anxMark microplate absorbance spectrophotometer (Bio-Rad Laboratories Inc., Hercules, CA, USA) at a wavelength of 640 nm. Cell viability was recorded by using the TC10 automated cell counter.

### 4.3. Time-Course Assay Using Cytokine IL-4 and IL-13

A time course assay was performed with an optimum concentration of n-3 and n-6 fatty acids. Once again, the cells were cultured in five (one for each time interval) 24-well plates at a density of ~50,000 cells per well. Cells were counted using the TC10 automated cell counter. HEK-Blue cells were pipetted into 15 mL tubes, which were centrifuged at 1200 rpm for 5 min. The supernatant was discarded and experimental media was added to suspend the cell pellet. If required, cell tires were reduced by adding growth medium to achieve optimum cell density. The cells were pipetted into 24-well plates (Corning, Sigma-Aldrich, St. Louis, MO, USA) at a density of ~50,000 cells per well in a total volume of 720 μL. Concentration of fatty acids used at this stage was 90 μM, which is within high physiological levels and the most optimum concentration of fatty acid to inhibit SEAP secretion (established from previous section). Cells were incubated in a humidified incubator at 37 °C with 5% CO_2_ for a period of 24 h. A total of five time-points were used to measure the effect of fatty acids on SEAP production over a period of 24 h; 0, 6, 12, 18 and 24 h, respectively. PC’s were also setup for each time interval, which was without fatty acid treatment. Post-incubation, 80 μL of cytokine IL-4 and IL-13 were pipetted to each treatment well at a concentration of 100 ng/mL and the plates were incubated for 24 h to stimulate the STAT6 phosphorylation pathway. Simultaneously, the experimental media in the wells was replaced with a fresh supply of growth medium to ensure a steady supply of nutrients to the cells. Twenty μL of induced HEK-Blue IL-4/IL-13 cell supernatant from each treatment well was added to a corresponding plate with 180 μL of Quanti-Blue substrate in each well. This was incubated for a total of 3 h to detect SEAP levels. The blue color was detected using a spectrophotometer at 640nm. Cell viability was recorded by using the TC10 automated cell counter.

### 4.4. Statistical Analysis

The data were analysed using Microsoft Excel 2013 (Microsoft Inc., Redmond, WA, USA). The results were analysed by Student’s *t*-test using a two-tailed method to determine any statistically significant difference between the controls and treatment wells in all of the above experiments. The statistical significance was set at *p* < 0.05.

## 5. Conclusions

Overall, treatment of HEK-Blue cells with n-3 fatty acids led to a suppression of SEAP production through inhibition of IL-4 and IL-13 signaling pathways. These results are biologically relevant to the development of a prophylactic treatment for allergic diseases. With n-6 fatty acids, on the other hand, an increase in SEAP output was observed, which is consistent with previous studies. Our study presented here opens a new corridor to research with n-3 fatty acids using a transfected model for *in vitro* allergy research. DHA, EPA and DPA supplementation may offer a molecular basis for a well-developed anti-allergic effect and, therefore, must be an essential part of our regular diet. Although further investigation will reveal the best allergy markers that can be used for suppression, it is clear that n-3 fatty acids may offer a great opportunity as potential natural dietary inhibitors for allergy.

## References

[1] Arshad S.H. (2005). Primary prevention of asthma and allergy. J. Allergy Clin. Immunol..

[2] Gergen P.J. (2001). Understanding the economic burden of asthma. J. Allergy Clin. Immunol..

[3] Lepski S., Brockmeyer J. (2013). Impact of dietary factors and food processing on food allergy. Mol. Nutr. Food Res..

[4] Tedeschi A., Barcella M., Dal Bo G.A., Miadonna A. (2003). Onset of allergy and asthma symptoms in extra-European immigrants to Milan, Italy: Possible role of environmental factors. Clin. Exp. Allergy.

[5] Wang D.Y. (2005). Risk factors of allergic rhinitis: Genetic or environmental?. Ther. Clin. Risk Manag..

[6] Graham-Rowe D. (2011). Lifestyle: When allergies go west. Nature.

[7] Klemens C.M., Berman D.R., Mozurkewich E.L. (2011). The effect of perinatal omega-3 fatty acid supplementation on inflammatory markers and allergic diseases: A systematic review. Int. J. Obstet. Gynaecol..

[8] Blumer N., Renz H. (2007). Consumption of omega3-fatty acids during perinatal life: Role in immuno-modulation and allergy prevention. J. Perinat. Med..

[9] Simopoulos A.P. (2002). Omega-3 fatty acids in inflammation and autoimmune diseases. J. Am. Coll. Nutr..

[10] Dunstan J.A., Mori T.A., Barden A., Beilin L.J., Taylor A.L., Holt P.G., Prescott S.L. (2003). Fish oil supplementation in pregnancy modifies neonatal allergen-specific immune responses and clinical outcomes in infants at high risk of atopy: A randomized, controlled trial. J. Allergy Clin. Immunol..

[11] Furuhjelm C., Warstedt K., Larsson J., Fredriksson M., Böttcher M.F., Fälth-Magnusson K., Duchén K. (2009). Fish oil supplementation in pregnancy and lactation may decrease the risk of infant allergy. Acta Paediatr..

[12] Lauritzen L., Kjær T.M.R., Fruekilde M.-B., Michaelsen K.F., Frøkiær H. (2005). Fish oil supplementation of lactating mothers affects cytokine production in 2 1/2-year-old children. Lipids.

[13] Gottrand F. (2008). Long-chain polyunsaturated fatty acids influence the immune system of infants. J. Nutr..

[14] Burks A.W., Tang M.M., Sicherer S., Muraro A., Eigenmann P.A., Ebisawa M., Fiocchi A., Chiang W., Beyer K., Wood R. (2012). ICON: Food allergy. J. Allergy Clin. Immunol..

[15] Ngoc P.L., Gold D.R., Tzianabos A.O., Weiss S.T., Celedón J.C. (2005). Cytokines, allergy, and asthma. Curr. Opin. Allergy Clin. Immunol..

[16] Weise C., Hilt K., Milovanovica M., Ernst D., Rühl R., Worm M. (2011). Inhibition of IgE production by docosahexaenoic acid is mediated by direct interference with STAT6 and NFkappaB pathway in human B cells. J. Nutr. Biochem..

[17] West C.E., Videky D.J., Prescott S.L. (2010). Role of diet in the development of immune tolerance in the context of allergic disease. Curr. Opin. Pediatr..

[18] Kremmyda L.S., Vlachava M., Noakes P.S., Diaper N.D., Miles E.A., Calder P.C. (2011). Atopy risk in infants and children in relation to early exposure to fish, oily fish, or long-chain omega-3 fatty acids: A systematic review. Clin. Rev. Allergy Immunol..

[19] Shek L.P., Chong M.F.-F., Lim J.Y., Soh S.E., Chong Y.S. (2012). Role of Dietary Long-Chain Polyunsaturated Fatty Acids in Infant Allergies and Respiratory Diseases. Clin. Dev. Immunol..

[20] Van den Elsen L., Garssen L.J., Willemsen L. (2012). Long chain N-3 polyunsaturated fatty acids in the prevention of allergic and cardiovascular disease. Curr. Pharm. Des..

[21] Rosell M.S., Lloyd-Wright Z., Appleby P.N., Sanders T.A.B., Allen N.E., Key T.J. (2005). Long-chain n-3 polyunsaturated fatty acids in plasma in British meat-eating, vegetarian, and vegan men. Am. J. Clin. Nutr..

[22] Johansson S., Wold A.E., Sandberg A.S. (2011). Low breast milk levels of long-chain n-3 fatty acids in allergic women, despite frequent fish intake. Clin. Exp. Allergy.

[23] Kull I., Bergström A., Lilja G., Pershagen G., Wickman M. (2006). Fish consumption during the first year of life and development of allergic diseases during childhood. Allergy.

[24] Alm B., Åberg N., Erdes L., Möllborg P., Pettersson R., Norvenius S.G., Goksör E., Wennergren G. (2009). Early introduction of fish decreases the risk of eczema in infants. Arch. Dis. Child..

[25] Kis L.L., Gerasimčik N., Salamon D., Persson E.K., Nagy N., Klein G., Severinson E., Klein E. (2011). STAT6 signaling pathway activated by the cytokines IL-4 and IL-13 induces expression of the Epstein-Barr virus–encoded protein LMP-1 in absence of EBNA-2: Implications for the type II EBV latent gene expression in Hodgkin lymphoma. Blood.

[26] Van Meter A.R., Ehringer W.D., Stillwell W., Blumenthal E.J., Jenski L.J. (1994). Aged lymphocyte proliferation following incorporation and retention of dietary omega-3 fatty acids. Mech. Ageing Dev..

[27] Koch C., Dölle S., Metzger M., Rasche C., Jungclas H., Rühl R., Renz H., Worm M. (2008). Docosahexaenoic acid (DHA) supplementation in atopic eczema: A randomized, double-blind, controlled trial. Br. J. Dermatol..

[28] Zhou L., Nilsson A. (2001). Sources of eicosanoid precursor fatty acid pools in tissues. J. Lipid Res..

[29] Simopoulos A.P. (1999). Essential fatty acids in health and chronic disease. Am. J. Clin. Nutr..

[30] Pla A.F., Grange C., Antoniotti S., Tomatis C., Merlino A., Bussolati B., Munaron L. (2008). Arachidonic Acid–Induced Ca^2+^ Entry Is Involved in Early Steps of Tumor Angiogenesis. Mol. Cancer Res..

[31] Inagaki N., Nagai H., Koda A. (1984). Effect of vitamin E on IgE antibody formation in mice. J. Pharmacobiodyn..

[32] Tsoureli-Nikita E., Hercogova J., Lotti T., Menchini G. (2002). Evaluation of dietary intake of vitamin E in the treatment of atopic dermatitis: A study of the clinical course and evaluation of the immunoglobulin E serum levels. Int. J. Dermatol..

[33] Stubbs C.D., Smith A.D. (1984). The modification of mammalian membrane polyunsaturated fatty acid composition in relation to membrane fluidity and function. Biochim. Biophys. Acta.

[34] Graber R., Sumida C., Nunez E.A. (1994). Fatty acids and cell signal transduction. J. Lipid Mediat. Cell Signal..

[35] Monick M.M., Robeff P.K., Butler N.S., Flaherty D.M., Flaherty A.B., Peterson M.W., Hunninghake G.W. (2002). Phosphatidylinositol 3-kinase activity negatively regulates stability of cyclooxygenase 2 mRNA. J. Biol. Chem..

[36] Miles E.A., Aston L., Calder P.C. (2003). *In vitro* effects of eicosanoids derived from different 20-carbon fatty acids on T helper type 1 and T helper type 2 cytokine production in human whole-blood cultures. Clin. Exp. Allergy.

[37] Bagga D., Wang L., Farias-Eisner R., Glaspy J.A., Reddy S.T. (2003). Differential effects of prostaglandin derived from ω-6 and ω-3 polyunsaturated fatty acids on COX-2 expression and IL-6 secretion. Proc. Natl. Acad. Sci. USA.

[38] Adegboyega P.A., Ololade O. (2004). Immunohistochemical expression of cyclooxygenase-2 in normal kidneys. Appl. Immunohistochem. Mol. Morphol..

[39] Harris R.C. (2000). Cyclooxygenase-2 in the Kidney. J. Am. Soc. Nephrol..

[40] Fedyk E.R., Phipps R.P. (1996). Prostaglandin E2 receptors of the EP2 and EP4 subtypes regulate activation and differentiation of mouse B lymphocytes to IgE-secreting cells. Proc. Natl. Acad. Sci. USA.

